# Toward Sharing Brain Images: Differentially Private TOF-MRA Images With Segmentation Labels Using Generative Adversarial Networks

**DOI:** 10.3389/frai.2022.813842

**Published:** 2022-05-02

**Authors:** Tabea Kossen, Manuel A. Hirzel, Vince I. Madai, Franziska Boenisch, Anja Hennemuth, Kristian Hildebrand, Sebastian Pokutta, Kartikey Sharma, Adam Hilbert, Jan Sobesky, Ivana Galinovic, Ahmed A. Khalil, Jochen B. Fiebach, Dietmar Frey

**Affiliations:** ^1^CLAIM-Charité Lab for AI in Medicine, Charité Universitätsmedizin Berlin, Berlin, Germany; ^2^Department of Computer Engineering and Microelectronics, Computer Vision & Remote Sensing, Technical University Berlin, Berlin, Germany; ^3^QUEST Center for Responsible Research, Berlin Institute of Health (BIH), Charité-Universitätsmedizin Berlin, Berlin, Germany; ^4^Faculty of Computing, Engineering and the Built Environment, School of Computing and Digital Technology, Birmingham City University, Birmingham, United Kingdom; ^5^Fraunhofer AISEC, Berlin, Germany; ^6^Institute for Imaging Science and Computational Modelling in Cardiovascular Medicine, Charité Universitätsmedizin Berlin, Berlin, Germany; ^7^Fraunhofer MEVIS, Bremen, Germany; ^8^Department VI Computer Science and Media, Berlin University of Applied Sciences and Technology, Berlin, Germany; ^9^Department for AI in Society, Science, and Technology, Zuse Institute Berlin, Berlin, Germany; ^10^Institute of Mathematics, Technical University Berlin, Berlin, Germany; ^11^Johanna-Etienne-Hospital, Neuss, Germany; ^12^Centre for Stroke Research Berlin, Charité Universitätsmedizin Berlin, Berlin, Germany; ^13^Department of Neurology, Max Planck Institute for Human Cognitive and Brain Sciences, Leipzig, Germany; ^14^Mind, Brain, Body Institute, Berlin School of Mind and Brain, Humboldt-Universität Berlin, Berlin, Germany

**Keywords:** brain vessel segmentation, differential privacy, Generative Adversarial Networks, neuroimaging, privacy preservation

## Abstract

Sharing labeled data is crucial to acquire large datasets for various Deep Learning applications. In medical imaging, this is often not feasible due to privacy regulations. Whereas anonymization would be a solution, standard techniques have been shown to be partially reversible. Here, synthetic data using a Generative Adversarial Network (GAN) with differential privacy guarantees could be a solution to ensure the patient's privacy while maintaining the predictive properties of the data. In this study, we implemented a Wasserstein GAN (WGAN) with and without differential privacy guarantees to generate privacy-preserving labeled Time-of-Flight Magnetic Resonance Angiography (TOF-MRA) image patches for brain vessel segmentation. The synthesized image-label pairs were used to train a U-net which was evaluated in terms of the segmentation performance on real patient images from two different datasets. Additionally, the Fréchet Inception Distance (FID) was calculated between the generated images and the real images to assess their similarity. During the evaluation using the U-Net and the FID, we explored the effect of different levels of privacy which was represented by the parameter ϵ. With stricter privacy guarantees, the segmentation performance and the similarity to the real patient images in terms of FID decreased. Our best segmentation model, trained on synthetic and private data, achieved a Dice Similarity Coefficient (DSC) of 0.75 for ϵ = 7.4 compared to 0.84 for ϵ = ∞ in a brain vessel segmentation paradigm (DSC of 0.69 and 0.88 on the second test set, respectively). We identified a threshold of ϵ <5 for which the performance (DSC <0.61) became unstable and not usable. Our synthesized labeled TOF-MRA images with strict privacy guarantees retained predictive properties necessary for segmenting the brain vessels. Although further research is warranted regarding generalizability to other imaging modalities and performance improvement, our results mark an encouraging first step for privacy-preserving data sharing in medical imaging.

## 1. Introduction

Deep Learning techniques are on the rise in many neuroimaging applications (Lundervold and Lundervold, 2019; Zhu et al., 2019; Hilbert et al., 2020). While showing great potential, they also demand large amounts of data. In medical imaging, data is often limited and medical experts are often needed to manually label the images (Willemink et al., 2020). Thus, large datasets are difficult to acquire. One potential solution would be data sharing. For this, true anonymization, i.e. verifying that no identifying information is leaked, is essential to sustain the patient's privacy which poses a big challenge, especially for neuroimaging (Bannier et al., 2021). For example, face-recognition software has recently identified individuals on medical images (Schwarz et al., 2019) and even face removal techniques can be partially reversed (Abramian and Eklund, 2019). Besides that, the brain itself has a unique structure and cortical foldings can be utilized to identify individuals even in the developing stage (Duan et al., 2020). Consequently, it is highly challenging to truly anonymize brain scans without risking re-identification. A promising remedy is the generation of synthetic data.

For this purpose, Generative Adversarial Networks (GANs) have gained a lot of attention in the past years (Yi et al., 2019). This also holds true for the neuroimaging domain. Here, GANs have shown promising results for synthesized images for different types of imaging (Bowles et al., 2018; Foroozandeh and Eklund, 2020; Kossen et al., 2021) as well as for other medical problems such as segmentation (Cirillo et al., 2020). To ensure the privacy of the training data, GANs can be combined with differential privacy (Xie et al., 2018). Differential privacy is a mathematical framework that provides an upper bound on individual privacy leakage (Dwork, 2008). This way the maximum privacy leakage for every individual in the training data can be quantified. There are extensive studies about GANs with differential privacy for synthesizing natural images and tabular medical data (Xie et al., 2018; Torkzadehmahani et al., 2019; Xu et al., 2019; Yoon et al., 2019, 2020). Recently, Cheng et al. (2021) did a comprehensive study about synthetic images and classification fairness with a varying amount of privacy on various types of imaging data. Among them were also 2D medical datasets such as chest x-rays and melanoma images. Few other studies generated chest x-rays with privacy guarantees as well (Nguyen et al., 2021; Zhang et al., 2021). However, to date, no study has investigated whether 2D synthesized data using a GAN with differential privacy can be utilized for a 3D medical application. Additionally, to the best of our knowledge, GANs with differential privacy have neither been used to synthesize labels for medical images nor the neuroimaging domain yet.

In this study, we utilized a Wasserstein GAN (WGAN) with and without differential privacy guarantees to synthesize anonymously and labeled 2D Time-of-Flight Magnetic Resonance Angiography (TOF-MRA) image patches for brain vessel segmentation. The generated labeled image patches were evaluated in terms of the segmentation performance by training a U-Net and in terms of image quality using the Fréchet Inception Distance (FID). The trained U-Net was further tested on a second dataset. Overall, we investigated the effect of different levels of privacy. Additionally, we visualized generated images with and without privacy together with the real patient images using t-distributed stochastic neighbor embedding (t-SNE).

In summary, our contributions are:

1. To the best of our knowledge, we are the first to synthesize images with differential privacy guarantees in the neuroimaging domain.
2. We also generate the corresponding segmentation labels to evaluate the image-label pairs in an end-to-end brain vessel segmentation paradigm on 3D medical data for different levels of privacy.
3. For evaluation, we compare the distances between the generated data and both the training and test data to investigate the similarity of the synthesized to the original data.
4. We visualize our generated images with and without differential privacy and the original data using t-SNE.

## 2. Related Study

For the synthesis of medical images, deep generative models have demonstrated promising results. Among them, especially GANs and variational autoencoders (VAE) have shown good performance in tasks such as data augmentation (Bowles et al., 2018), image-to-image translations (Isola et al., 2018), or reconstruction (Tudosiu et al., 2020). For the purpose of synthesizing privacy-preserving images, VAE has two disadvantages compared to GANs: First, they produce blurrier images (Wang et al., 2020), and second, the training images are directly fed into the network which makes them more vulnerable to membership inference attacks (Chen et al., 2020).

Hence, in this context, GAN architectures with differential privacy have been used in many previous studies to synthesize non-medical images (Xie et al., 2018; Torkzadehmahani et al., 2019; Xu et al., 2019) and medical tabular data (Yoon et al., 2019, 2020). However, only few studies have applied GANs with differential privacy to medical images. Additionally, these were restricted to chest x-rays (Cheng et al., 2021; Nguyen et al., 2021; Zhang et al., 2021). So far in the neuroimaging domain, the application of GANs remained without differential privacy (Bowles et al., 2018; Foroozandeh and Eklund, 2020; Kossen et al., 2021).

In the present study, we propose a GAN architecture with differential privacy in the neuroimaging domain. Along with our synthesized images, we generate the segmentation labels for testing our differentially private patches in an end-to-end brain vessel segmentation paradigm.

## 3. Materials and Methods

### 3.1. Data

In total, 131 patients with cerebrovascular disease from the PEGASUS study (N = 66) and the 1000Plus study (N = 65) were utilized in this study. All patients gave their written informed consent and the studies have been authorized by the ethical review committee of Charité–Universitätsmedizin Berlin. More details on both datasets can be found in Mutke et al. (2014) for the PEGASUS study and Hotter et al. (2009) for the 1000Plus study.

The brain scans were conducted on a clinical 3T whole-body system (Magnetom Trio, Siemens Healthcare, Erlangen, Germany) utilizing a 12-channel receive radiofrequency coil (Siemens Healthcare) for head imaging. For both studies the parameters were: voxel size = (0.5 x 0.5 x 0.7) mm^3^; matrix size: 312 x 384 x 127; TR/TE = 22 ms/3.86 ms; acquisition time: 3:50 min, flip angle = 18°.

**Figure 1:**
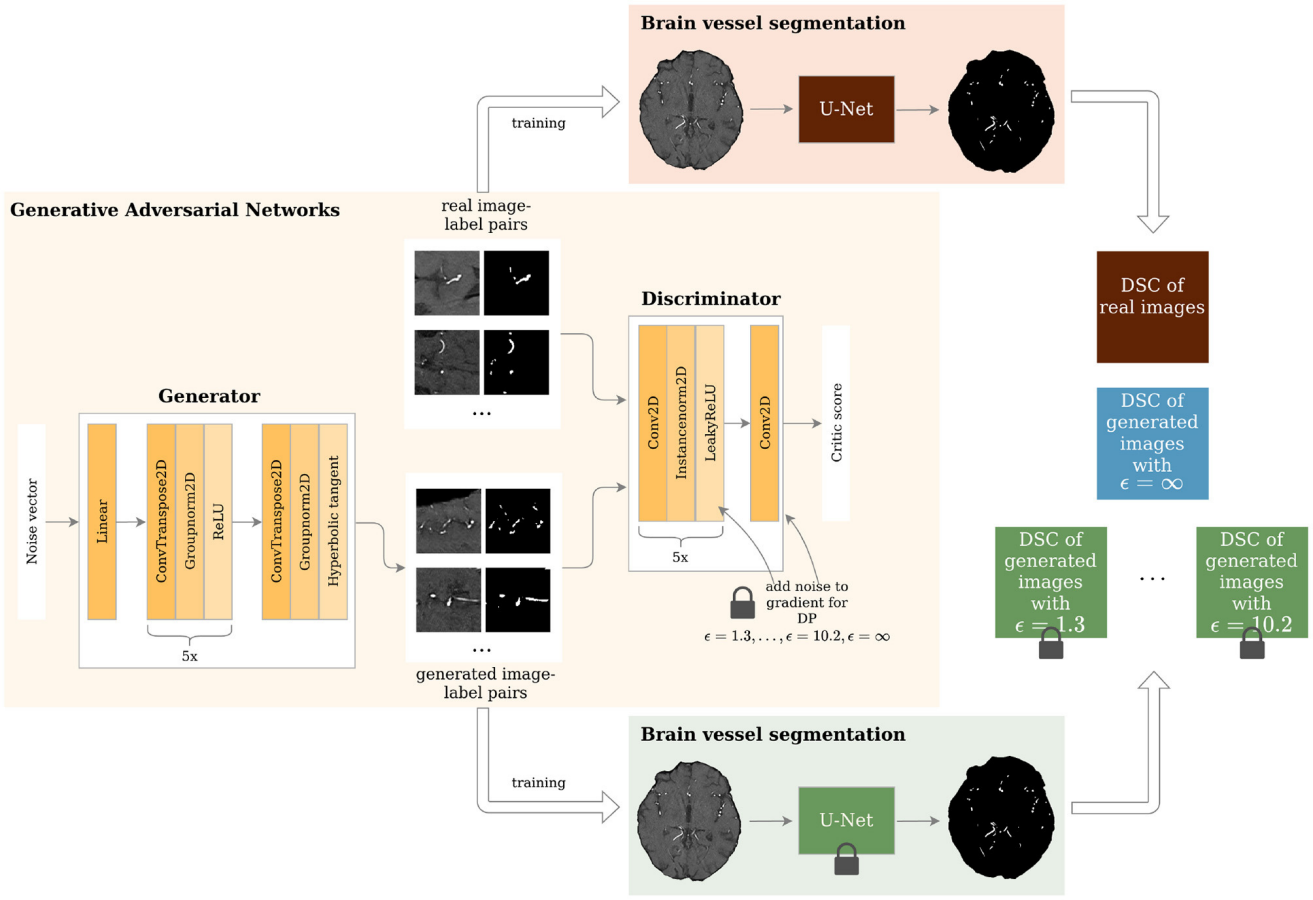
Study overview. Generative Adversarial Networks (GANs) with different levels of privacy guarantees are trained to synthesize labeled Time-of-Flight Magnetic Resonance Angiography (TOF-MRA) patches. These are evaluated in a brain vessel segmentation paradigm and are compared to a segmentation network trained on real patient image-label pairs. DP = Differential Privacy; DSC = Dice Similarity Coefficient.

The PEGASUS dataset was split into a training (41 patients), validation (11 patients), and test (14 patients) set. The training set was utilized for training the GANs (refer to Figure 1), whereas the validation and test set were utilized for the parameter selection of the U-Net and assessing the generalizable performance of the U-Net, respectively. Additionally, the 65 patients from the 1000Plus dataset were used as a second test set.

For each patient of the training set 1,000 2D image patches and corresponding segmentation masks of size 96x96 were extracted. This patch size has been shown to be the most suitable patch size for Wasserstein based GAN architectures for this use case (Kossen et al., 2021). Due to the overemphasis of background compared to brain vessels, 500 patches showing a vessel in the center were extracted. The remaining 500 patches were extracted randomly. It was verified that all patches were only selected at most once.

### 3.2. Differential Privacy

To account for the level of privacy of the generated data and provide theoretical privacy guarantees, differential privacy was implemented (Dwork, 2008). A randomized algorithm *f*:*d* → *R* satisfies (ϵ, δ)-differential privacy if for any two databases *d*_1_, *d*_2_ ∈ *d* that differ from each other by a single sample, the following holds:


(1) $$\begin{array}{l} \text{Pr}\left[f\left(d_{1}\right)\in S\right]\leq \exp\left(\epsilon \right)*\text{Pr}\left[f\left(d_{2}\right)\in S\right]+\delta \end{array}$$

where *f*(*d*_1_) and *f*(*d*_2_) denote the output of *f* and Pr the probabilities and with *S* ⊂ *R*. δ is the probability that the value of ϵ holds true. With a probability of 1 − δ this equation is equivalent to:


(2) $$\begin{array}{l} \log\left(\frac{\text{Pr}\left[f\left(d_{1}\right)\in S\right]}{\text{Pr}\left[f\left(d_{2}\right)\in S\right]}\right)\leq \epsilon . \end{array}$$

Thus, differential privacy holds true if the algorithm's output for *d*_1_ and *d*_2_ is very similar to each other. In other words, one sample should not have a big impact on the algorithm's output. This way the privacy of each possible datapoint is preserved. The maximal deviation between the outputs is given by exp(ϵ). In this way, ϵ can quantify the level of privacy with small values of ϵ indicating stricter privacy guarantees.

Mironov (2017) proposed Rényi differential privacy, a natural relaxation of differential privacy built upon Rényi divergence. Rényi divergence of order α>1 of two probability distributions *P* and *Q* is defined as:


(3) $$\begin{array}{l} D_{\alpha }\left(P\|Q\right):=\frac{1}{\alpha -1}\log E_{x\sim Q}{\left(\frac{P\left(x\right)}{Q\left(x\right)}\right)}^{\alpha }, \end{array}$$

where *P*(*x*) is the probability density of *P* at point *x*. A randomized algorithm *f*:*d* → *S* is (α, ϵ)-Rényi differentially private for any adjacent *d*_1_, *d*_2_ ∈ *d* if the Rényi divergence *D*_α_ is not larger than ϵ:


(4) $$\begin{array}{l} D_{\alpha }\left(f\left(d_{1}\right)\|f\left(d_{2}\right)\right)\leq \epsilon . \end{array}$$

The advantage of Rényi differential privacy is that it provides a tight composition for Gaussian mechanisms while preserving essential properties of differential privacy. This means that (α, ϵ)-Rényi differential privacy for composed mechanisms add up: the composition of *f*(*d*_1_) satisfying (α, ϵ_1_)-Rényi differential privacy and *f*(*d*_2_) satisfying (α, ϵ_2_)-Rényi differential privacy satisfies (α, ϵ_1_ + ϵ_2_)-Rényi differential privacy. Moreover, (α, ϵ)-Rényi differential privacy has been shown to provide a tighter bound on the privacy budget of compositions compared to (ϵ, δ)-differential privacy (Mironov, 2017). (α, ϵ)-Rényi differential privacy can also be translated back into (ϵ, δ)-differential privacy. Balle et al. (2019) has proven that (α, ϵ)-Rényi differential privacy also satisfies (ϵ′, δ)-differential privacy for any 0 < δ < 1. According to Balle et al. (2019) ϵ′ is then defined as:


(5) $$\begin{array}{l} \epsilon^{\prime }=\epsilon +\log\frac{\alpha -1}{\alpha }-\frac{\log\delta +\log\alpha }{\alpha -1}. \end{array}$$

The most data sensitive part when training the proposed GAN architecture is the gradient update of the discriminator after training samples are presented. For that, the differentially private stochastic gradient descent algorithm proposed by Abadi et al. (2016) can be utilized. Here, differential privacy was implemented by clipping these gradients and adding Gaussian noise to avoid the memorization of single samples. Additionally, Rényi differential privacy was then used to analyze the privacy guarantees. In the last step, (α, ϵ)-Rényi differential privacy is translated back to (ϵ, δ)-differential privacy. The parameter δ is typically chosen to be the inverse of the dataset size (Torkzadehmahani et al., 2019). Thus, throughout this study, it was set to 1/41, 000 = 2.44*e* − 5.

### 3.3. Network Architecture

The GAN architecture was based on the WGAN by Arjovsky et al. (2017) and extended by inserting different amounts of noise into the gradients of the discriminator in the training process for differential privacy. Two neural networks were trained: the generator *G* and the discriminator *D*. The generator synthesized data samples that were then assessed with respect to their realness by the critic or discriminator. The discriminator was fed both real and synthesized data and assigned a critic score for each sample. The score of the synthetic data *x*_gen_ was used to train the generator. For the generator the overall training loss was:


(6) $$\begin{array}{l} {\text{loss}}_{G}=-D\left(x_{\text{gen}}\right). \end{array}$$

This way the generator aimed to maximize the realness of the generated samples. In contrast to that, the discriminator intended to minimize the scores for generated samples *x*_gen_ and maximize them for patient samples *x*_real_:


(7) $$\begin{array}{l} {\text{loss}}_{D}=D\left(x_{\text{gen}}\right)-D\left(x_{\text{real}}\right) \end{array}$$

To enforce a Lipschitz constraint and, thus, put a bound on the gradients, the discriminator's weights were clipped after each backpropagation step. This is a simple way to stabilize the training (Arjovsky et al., 2017).

The architecture of the generator and discriminator is shown in Figure 1. The generator took a noise vector sampled from a Gaussian distribution of size 128 as input. This was then fed through 1 linear layer and 6 upsampling convolutional layers as shown in Figure 1. The generator outputs 2 96 x 96 images - 1 channel for the image and 1 for the segmentation label. The discriminator's input was 2 images: either the real patient image-label pair or the generated one. These were then fed through 6 layers of downsampling convolutional layers as depicted in Figure 1. The slope of the LeakyReLU activation was 0.2.

The GANs were implemented in PyTorch 1.8.1 using the library opacus 0.14.0 for the differential privacy guarantees. Our code was built upon the official GAN example by opacus^1^ and is publicly available^2^. The learning rate for both discriminator and generator was 0.00005 using the RMSprop optimizer. The kernel size was 4 with strides of 2. In each epoch, the discriminator was updated 5 times. The network was trained for 50 epochs. To randomly sample the training images, the UniformWithReplacementSampler from the opacus package was used. The sampling rate was the batch size of 32 divided by the number of samples (41,000). The clipping parameter for the WGAN was set to 0.01 and the clipping parameter for the differential privacy was 1. In total, 8 different GANs were trained with varying values of ϵ (noise multiplier was set to {∞, 2, 1.5, 1.2, 1, 0.8, 0.725, 0.65}). Each GAN trained with additional noise was trained 5 times for robust results.

All hyperparameters mentioned in the last paragraph were the result of a tuning process and all models were trained on a Tesla V100. The training time of one GAN including evaluation took ~1.4 days.

### 3.4. Performance Evaluation

Among the many metrics to evaluate synthetic data (Yi et al., 2019), we selected three to estimate the quality of our synthesized images. First, we evaluated our synthesized image-label pairs by visual inspection, and second, using the downstream task of segmentation as suggested by Yi et al. (2019). Additionally, we compared the images using the FID as proposed in previous studies (Haarburger et al., 2019; Coyner et al., 2022).

The generated image-label pairs were evaluated by a U-Net for brain vessel segmentation adapted from Livne et al. (2019). After training the GANs, 41,000 image-label pairs were generated. These were used to train 8 U-Net with different hyperparameter settings varying in learning rates, dropout, and classical data augmentation. The best U-Net was then selected based on the best Dice Similarity Coefficient (DSC) on the validation set that included real patient images. The final performance was then evaluated in terms of DSC and balanced average Hausdorff distance (bAHD) on the test set. The DSC that evaluated the segmented voxels is defined as:


(8) $$\begin{array}{l} \text{DSC}=\frac{2\text{TP}}{2\text{TP}+\text{FP}+\text{FN}} \end{array}$$

where TP are the true positives, FP are the false positives, and FN are the false negatives. As the DSC quantifies the overlap of the ground truth and prediction scaled by the total number of voxels in ground truth and prediction, it is a robust performance measure for imbalanced segmentations, i.e., images contain more background than segmented area. The bAHD is a newly proposed metric for evaluating segmentations (Aydin et al., 2021):


(9) $$\begin{array}{l} \text{bAHD}=\left(\frac{1}{N_{G}}\underset{g\in G}{\sum }\underset{s\in S}{\min}d\left(g,s\right)+\frac{1}{N_{G}}\underset{s\in S}{\sum }\underset{g\in G}{\min}d\left(s,g\right)\right)/2 \end{array}$$

where *N*_*G*_ is the number of ground truth voxels, *G* is the set of voxels belonging to the ground truth, and *S* is the set of voxels of the predicted segmentation. In other words, the bAHD is the average of the directed Hausdorff distance from the ground truth to the segmentation and the directed Hausdorff distance from the segmentation to the ground truth both scaled by the number of ground truth voxels.

Additionally, the DSC and bAHD of the U-Net models were assessed on the 1000Plus dataset. The GAN and U-Nets were implemented in an end-to-end pipeline. To calculate both DSC and bAHD, we used the EvaluateSegmentation tool by Taha and Hanbury (2015).

As an additional metric, the image quality was measured by the FID (Heusel et al., 2018). The FID is a distance that measures the similarity between images by comparing the activations of a pre-trained Inception-v3 network. Here, the difference between the activations in the pool3 layer of the generated images in contrast to the real images is measured.


(10) $$\begin{array}{l} \text{FID}=\|\mu_{\text{real}}-\mu_{\text{gen}}{\|}^{2}+\text{Tr}\left(\sigma_{\text{real}}+\sigma_{\text{gen}}-2{\left(\sigma_{\text{real}}\sigma_{\text{gen}}\right)}^{1/2}\right) \end{array}$$

with $N\left(\mu_{\text{real}},\sigma_{\text{real}}\right)$ and $N\left(\mu_{\text{gen}},\sigma_{\text{gen}}\right)$ as the distributions of the features of the pool3 layer of real and synthesized data, respectively.

To explore to which degree the generated images reproduced the training set, the FID between the synthetic data and both the training and test data was calculated and compared for different levels of privacy.

Finally, we measured the similarity between the images synthesized by the GANs to check whether a model suffered from mode collapse. For each model, we generated 1,000 images and calculated the Structural Similarity Index Measure (SSIM) between them and averaged the values. We repeated this analysis for all 5 runs for each ϵ value, for the model with ϵ = ∞ and the real images. The SSIM between two images *x* and *y* is defined as a product of luminance, contrast, and structure according to Wang et al. (2004):


(11) $$\begin{array}{l} \text{SSIM}\left(x,y\right)=\frac{\left(2\mu_{x}\mu_{y}+c_{1}\right)\left(2\sigma_{xy}+c_{2}\right)}{\left(\mu_{x}^{2}+\mu_{y}^{2}+c_{1}\right)\left(\sigma_{x}^{2}+\sigma_{y}^{2}+c_{2}\right)}, \end{array}$$

where μ_*x*_ is the average of *x*, σ_*x*_ is the variance, and σ_*xy*_ is the covariance of *x* and *y*. $c_{1}={\left(k_{1}L\right)}^{2}$ and $c_{2}={\left(k_{2}L\right)}^{2}$ are for stabilization with *L* being the dynamic range of the pixel values and *k*_1_ ≪ 1 and *k*_2_ ≪ 1 small constants.

### 3.5. Visualization Using t-SNE

Finally, the generated images with and without differential privacy and the real patient images were visualized using a t-SNE (Maaten and Hinton, 2008). t-SNE is an approach to reducing dimensionality while preserving the structure of the high dimensional data points. First, all data points are embedded into an SNE which computed the pairwise similarities utilizing conditional probabilities. For points *x*_*i*_ and *x*_*j*_ the conditional probability *p*_*j*|*i*_ of *x*_*i*_ choosing *x*_*j*_ as its neighbor is defined as


(12) $$\begin{array}{l} p_{j|i}=\frac{\exp(-{\left\|x_{i}-x_{j}\right\|}^{2}/2\sigma_{i}^{2})}{\sum_{k\neq i}\exp-{\left\|x_{i}-x_{k}\right\|}^{2}/2\sigma_{i}^{2})} \end{array}$$

and the symmetrized similarity as:


(13) $$\begin{array}{l} p_{ij}=\frac{p_{j|i}+p_{i|j}}{2N} \end{array}$$

with *N* being the dimensionality of the data. Then the algorithm aims to learn a lower dimensional representation of the similarities. In order to get distinct clusters and avoid overcrowding, a Student's *t* distribution that reflects the similarities *p*_*j*|*i*_ is used (Maaten and Hinton, 2008):


(14) $$\begin{array}{l} q_{ij}=\frac{{\left(1+\|y_{i}-y_{j}{\|}^{2}\right)}^{-1}}{\sum_{k\neq m}{\left(1+\|y_{k}-y_{m}{\|}^{2}\right)}^{-1}} \end{array}$$

Starting from random initialization, the locations of the points in the lower dimensional space *y*_*i*_ are shifted so that a cost function was minimized using a gradient descent method. Instead of the Kullback-Leibler divergence, we here chose the Wasserstein metric due to its success in GAN applications (Arjovsky et al., 2017).

T-distributed stochastic neighbor embedding was implemented using the sklearn package (Pedregosa et al., 2011). The perplexity parameter reflecting the density of the data distribution was chosen to be 30 which is in the suggested range by Maaten and Hinton (2008). The images of the best performing GAN with and without differential privacy, as well as the real images were projected, onto 2 dimensions for visualization purposes.

## 4. Results

**Figure 2:**
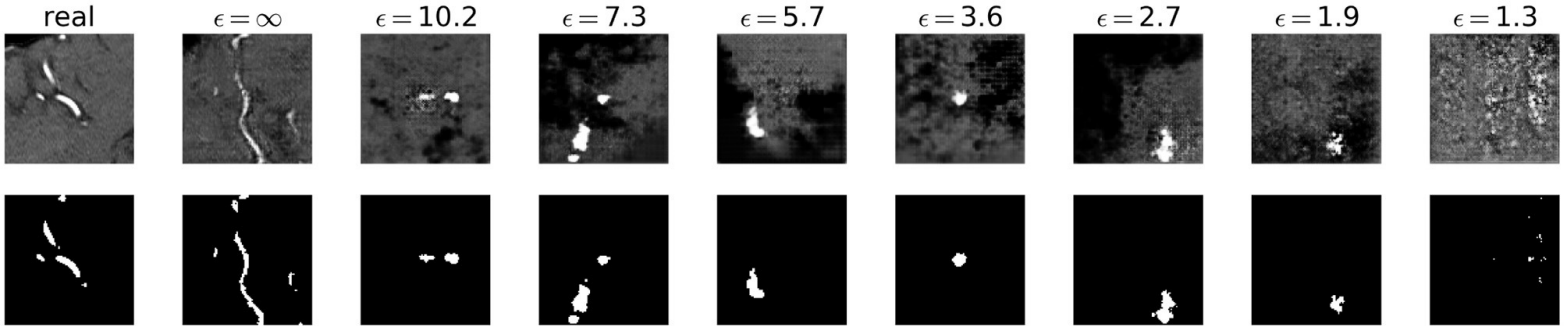
Synthetic TOF-MRA patches (top row) and corresponding segmentation labels (bottom row) with different values of ϵ compared to real patient data (first column). A lower ϵ (i.e., more privacy) leads to more noisy images.

**Figure 3:**
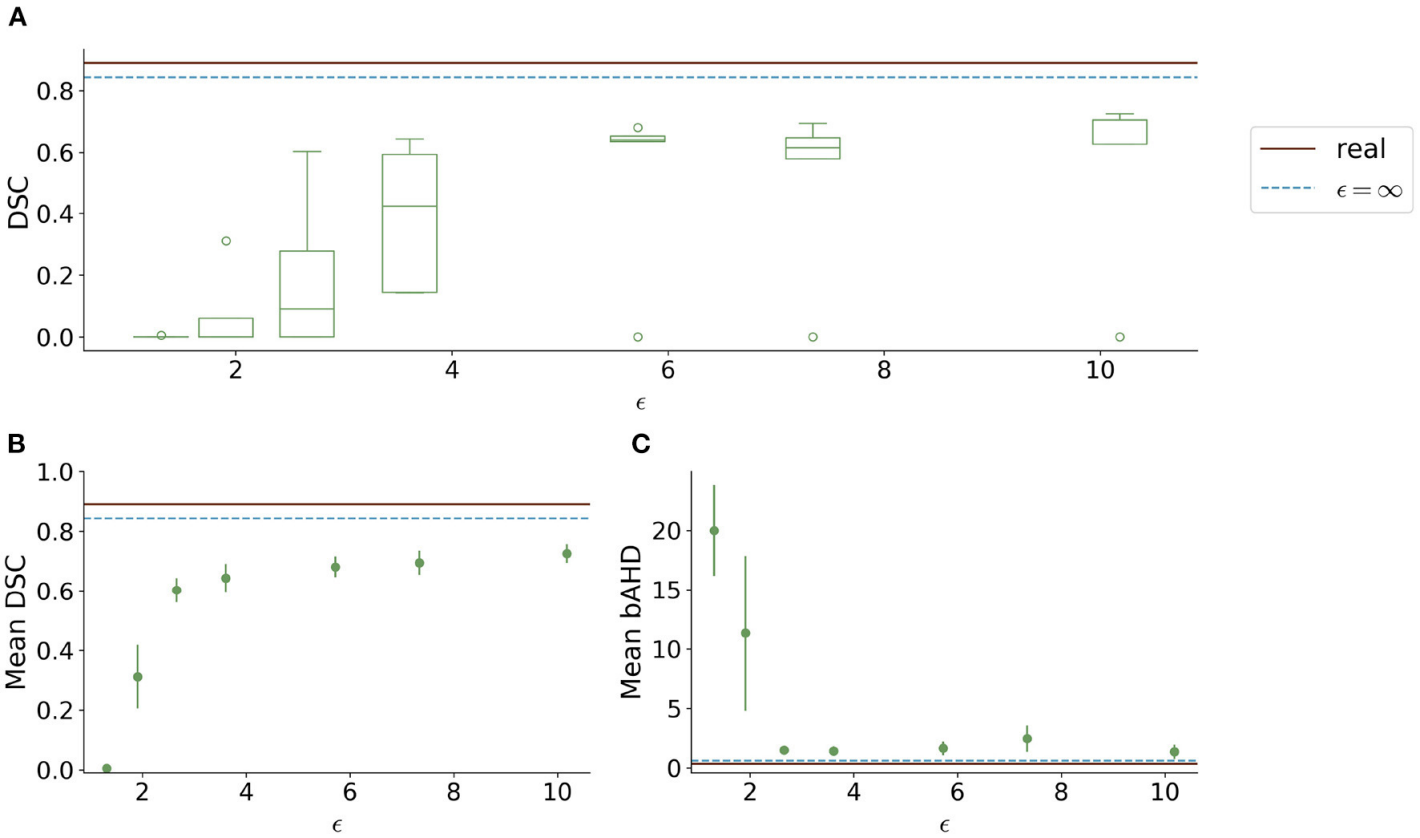
Test segmentation performance of U-Nets trained on generated data with different values of ϵ (PEGASUS dataset). **(A)** shows a boxplot showing the DSC over 5 runs for each value of ϵ. In **(B)**, only the run with the best DSC is shown. **(C)** shows the balanced average Hausdorff distance (bAHD) in voxels for the best run for each ϵ. The errorbar depicts the SD between patients. For ϵ < 5, the performance becomes unstable and worse compared to higher ϵ values.

**Figure 4:**
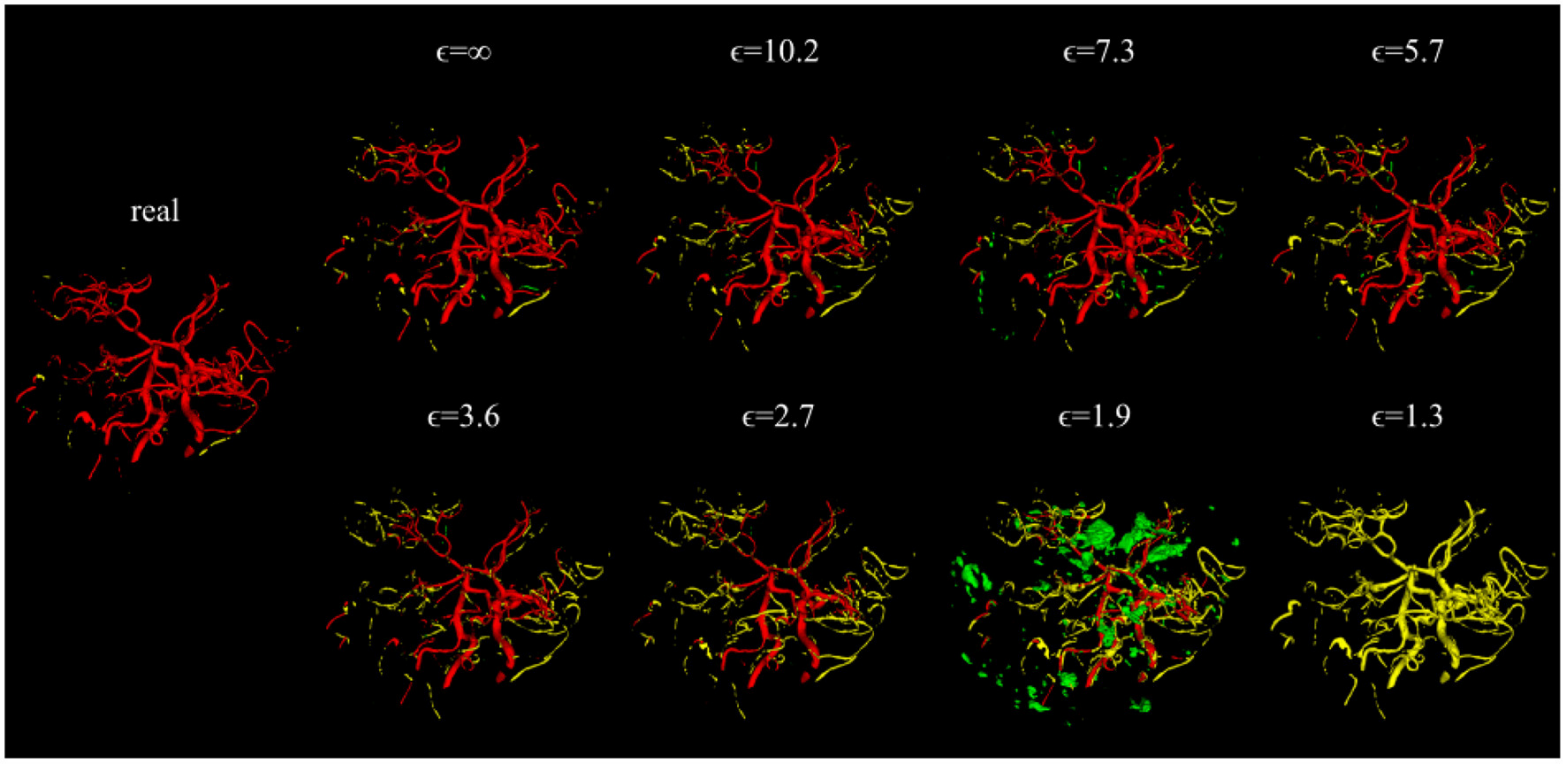
Error maps of one example test patient for U-Nets trained on either real image-label pairs or generated image-labels with different values of ϵ. True positives are shown in red, false positives in green, and false negatives in yellow. For lower ϵ, more errors occur.

Visually, the synthetic image-label pairs appeared noisier with decreasing ϵ, i.e., with stricter privacy guarantees (Figure 2). Differentially private images with ϵ = 1.3 show almost only noise. The visual results corresponded to the segmentation performance when training a U-Net on the generated image-label pairs with different values of ϵ (Figure 3). In Figure 3A, the averaged DSC over U-Net models that were trained on synthetic data from five different GANs for each ϵ is plotted. With decreasing ϵ, the DSC decreased and got more unstable, i.e., more variation between the different models for the same ϵ. In particular, models with ϵ>5 showed increased stability compared to models with lower ϵ. When considering only the best run of the five models (Figures 3B,C) the performance again dropped for decreasing ϵ. This was reflected by a lower DSC and a higher bAHD. The corresponding segmentation error maps are shown in Figure 4.

**Figure 5:**
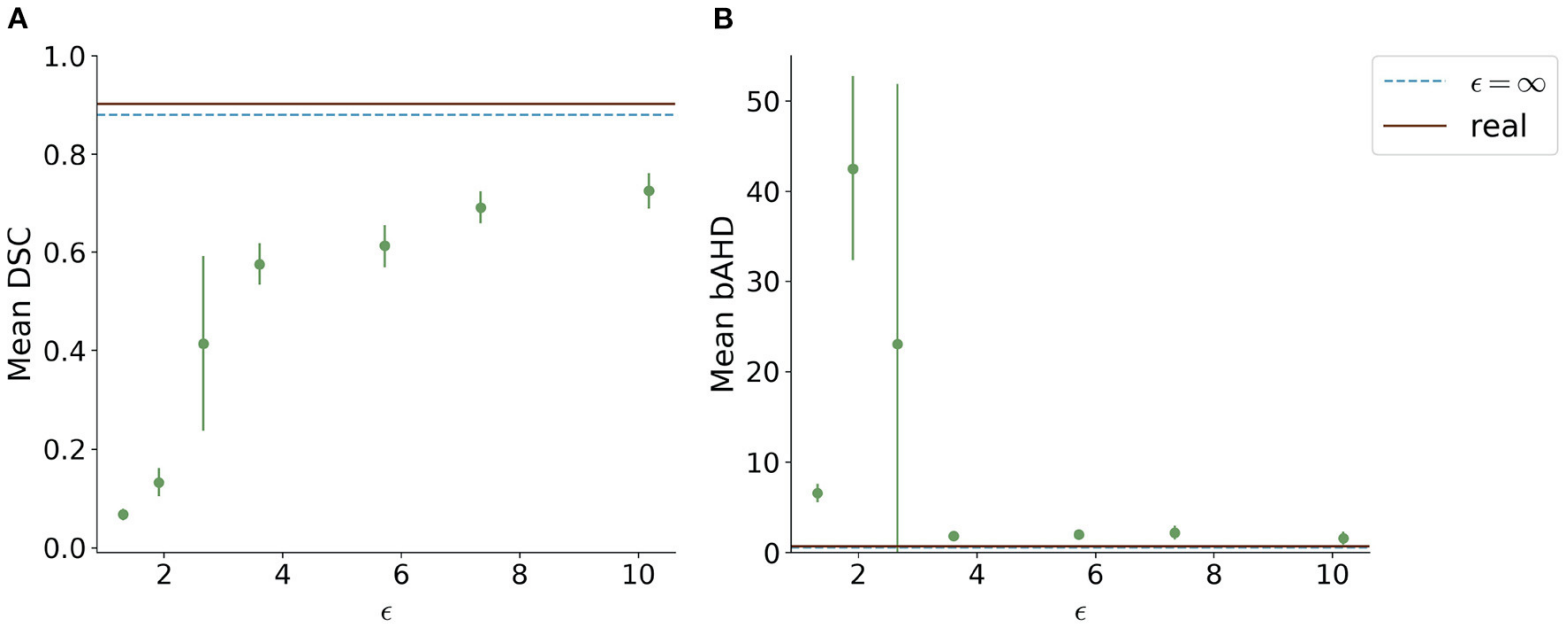
Segmentation performance in terms of **(A)** DSC and **(B)** bAHD in voxels of the best performing model for each ϵ evaluated on a second dataset (1000Plus). The DSC shows a decreasing performance starting for ϵ < 8.

When testing the best U-Net models on the 1000Plus dataset, a similar trade-off between privacy and utility can be seen (Figure 5). Here, the U-Net performance in terms of DSC decreased more rapidly in comparison to the performance on the PEGASUS dataset, starting at ϵ = 8 with DSC ≈0.69 (Figure 5A). The bAHD showed instability in performance for ϵ < 3 (Figure 5B).

**Figure 6:**
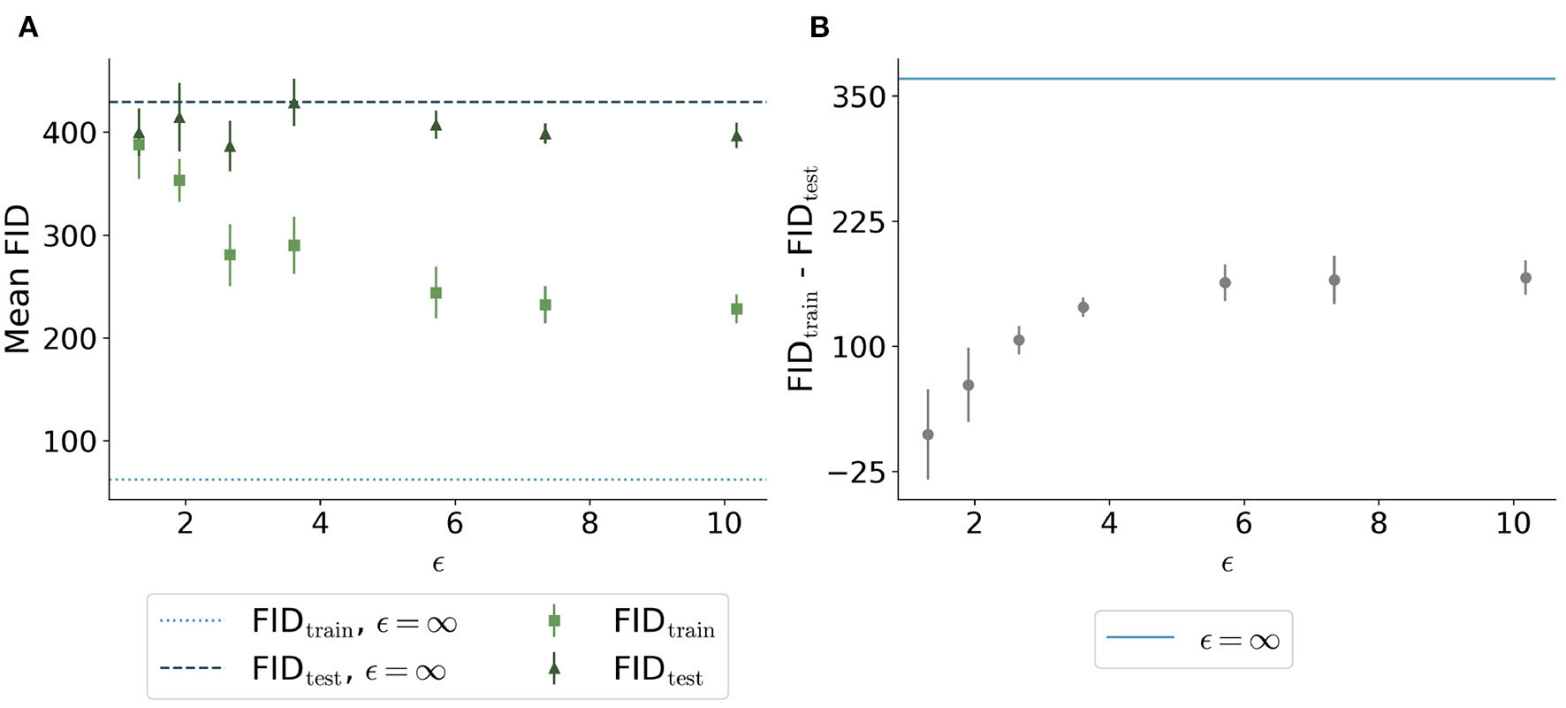
Comparison of Fréchet Inception Distance (FID) between the synthetic images with different ϵ values and both the real training data (light green squares and light blue dotted line) and the real test data (dark green triangles and dark blue dashed line). **(A)** shows the absolute values for the 5 runs per ϵ whereas **(B)** shows the difference between the distances from synthetic to training and synthetic to test. The higher the value of ϵ, the closer the images are to the training set. The distance to the test set remains stable for different ϵ values. The difference shown in **(B)** is the highest for the model trained without differential privacy.

The FID between the training data and the generated data overall showed a similar trend: Less privacy led to a smaller distance to the training data (Figure 6A). The generated data trained without differential privacy (ϵ = ∞) showed an FID of 62 compared to an FID of 244 and 228 for the images with ϵ = 5.7 and ϵ = 10.2, respectively. The distance to the test data was similar for different ϵ values. Figure 6B shows the difference between the distances to the training images and test images for different values of ϵ. Here, the differences were increasing for higher ϵ values with ϵ = ∞ showing the largest difference, at least twice as large compared to all models trained with privacy guarantees.

**Figure 7:**
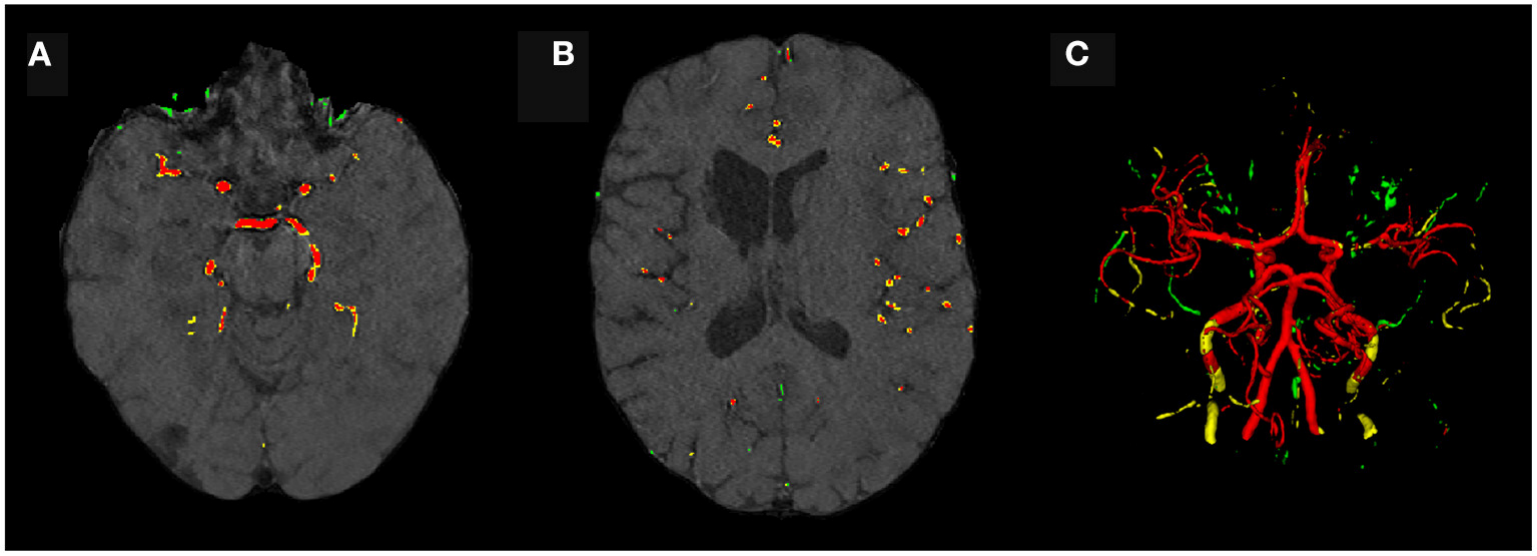
Segmentation error maps of one test patient by the best U-Net model using differential privacy (ϵ = 7.4). Red indicates the true positives, green stands for false positives, and yellow for false negatives. **(A)** shows a slice containing big vessels, **(B)** small ones, and **(C)** the whole vessel tree. The segmentation works reasonably well with errors occurring particularly when segmenting small vessels.

Evaluating GAN models during training, we found the best performing image-label pairs when training with a noise multiplier of 0.65 for 29 epochs. This resulted in ϵ = 7.4. The U-Net trained on these synthetic image-labels showed a DSC of 0.75 on the test set (Table 1). The segmentation of an example patient is shown in Figure 7. The big vessels are segmented reasonably well while a lot of errors occur when smaller vessels are segmented.

**Table 1:** Overview of segmentation performances in terms of DSC and bAHD for a U-Net trained on real patient images and generated with and without differential privacy. The best of the three U-Net models is shown in bold for each metric and dataset. The best U-Net with differential privacy guarantees has an ϵ of 7.4. SD stands for standard deviation.

	**PEGASUS**		**1000Plus**	
**U-Net trained on**	**Mean DSC (SD)**	**Mean bAHD (SD)**	**Mean DSC (SD)**	**Mean bAHD (SD)**
Real images	**0.89 (0.02)**	**0.33 (0.11)**	**0.90 (0.02)**	0.69 (0.47)
Generated images (ϵ = ∞)	0.84 (0.02)	0.61 (0.12)	0.88 (0.02)	**0.58 (0.32)**
Generated images (ϵ = 7.4)	0.75 (0.04)	2.49 (1.96)	0.69 (0.04)	2.87 (1.25)

**Figure 8:**
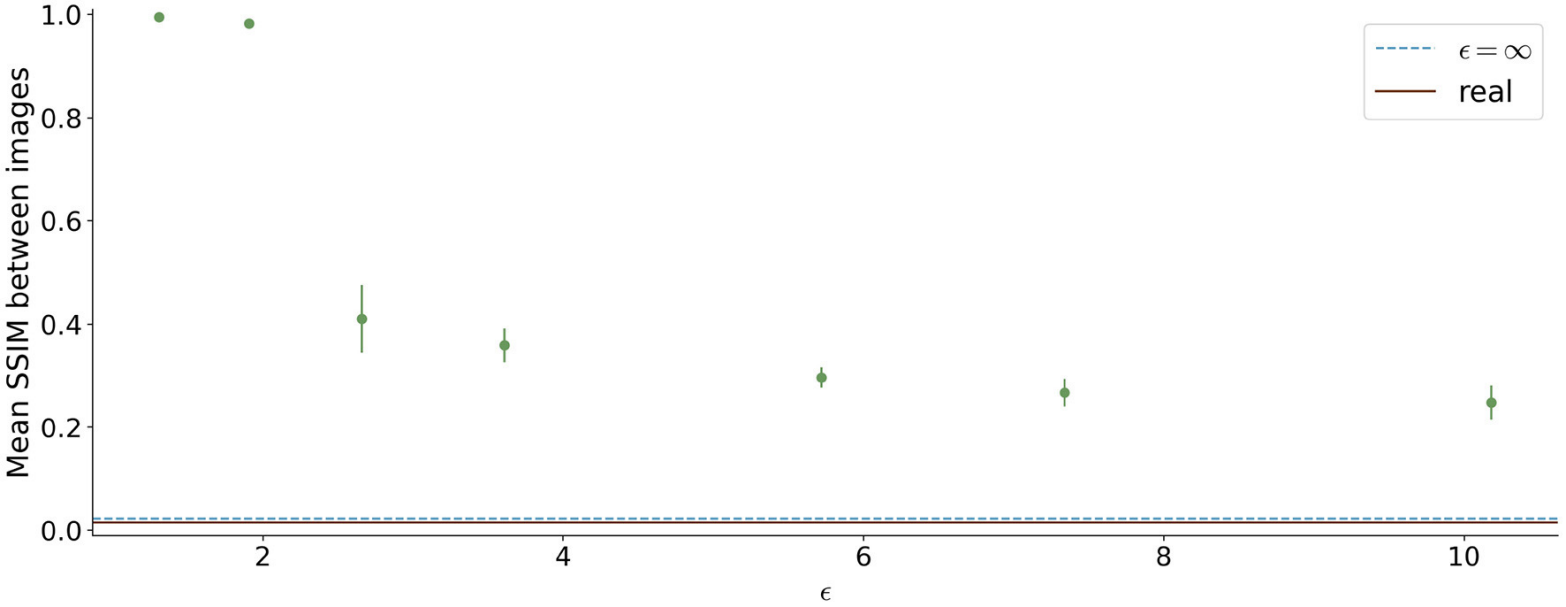
Mean Structural Similarity Index Measure (SSIM) between 1,000 generated images for differential ϵ values. The errorbar shows the standard deviation over the 5 different runs for each ϵ value. For ϵ < 2, the similarity between images is high, whereas it decreases for higher ϵ values.

The similarity between the images is shown in Figure 8. For ϵ < 2, high SSIM values were observed (SSIM > 0.98). In contrast, higher ϵ values led to less similar images produced by one model.

**Figure 9:**
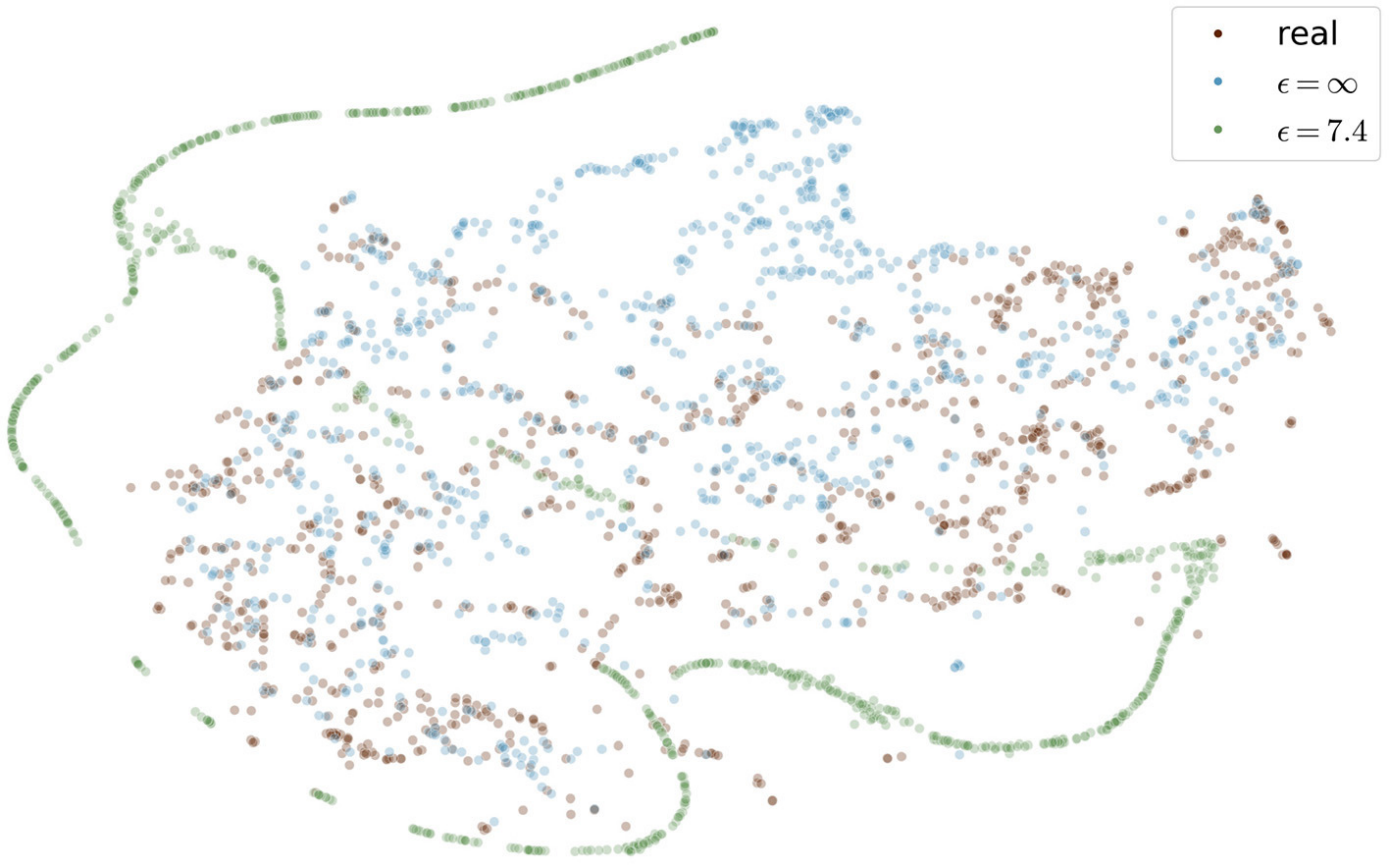
Visualization of real and generated images with and without differential privacy in a t-SNE embedding. Each point represents an image. The distribution of real images and generated images without privacy almost entirely overlap. In contrast, the images with privacy guarantees are only partially overlapping and cluster at the edges, distant from the real images. The embedding showing the specific image instead of a point can be found in the Figure S1 in the supplementary material.

Figure 9 shows the t-SNE embedding of the best performing GAN with and without differential privacy and the real patient images. The synthetic images without privacy guarantees are overall close to the real images. The images with differential privacy cluster at the edges far away from the real images.

## 5. Discussion

In the present study, we generated differentially private TOF-MRA images with corresponding labels and explored the trade-off between privacy and utility on two different test sets. We proposed different evaluation schemes including training a segmentation network and identified a threshold of ϵ < 5 with DSC < 0.61 for which the segmentation performance became unstable and not usable. Our best segmentation model trained on synthetic and private data achieved a DSC of 0.75 for ϵ = 7.4 in a brain vessel segmentation paradigm. Our results mark the first step in data sharing with privacy guarantees for neuroimaging problems.

Since differential privacy is based on introducing noise, a decrease in utility is expected with the introduction of differential privacy. Our results confirm this notion. For ϵ = ∞, we achieved a DSC of 0.84 which is comparable to the literature (Kossen et al., 2021). Stricter privacy constraints indicated by a lower ϵ led to worse visual results as well as poorer segmentation results (Figures 2–5). This also corresponds to findings in previous studies on differential privacy (Xie et al., 2018; Xu et al., 2019; Yoon et al., 2019). The increasing amount of noise might also be the reason for the instability of the GAN training for lower ϵ values, especially for ϵ < 5 (Figure 2A). A performance drop could also be observed for testing the U-Nets trained on differential private image-label pairs on a second dataset (Figure 5). In comparison to the first test set, the performance drop occurred already for higher values of ϵ (ϵ < 8 compared to ϵ < 5). Thus, models with fewer privacy guarantees showed better generalizability. A reason for that might be again the lower amount of noise and, therefore, fewer restrictions during training. This is also in line with our findings in Figure 8. Here, images generated from models with lower ϵ (ϵ < 2) values showed more similarities between each other, thus indicating more mode collapse compared to models with higher ϵ values. This could be another reason for the performance drop for models with stricter privacy guarantees.

Images with larger ϵ values also showed greater similarity in terms of FID to the training images than those with stricter privacy guarantees. This indicates that more specific features of the training set can be memorized for less noisy models. The FID between test images and synthetic images (FID_test_) stayed constant for different values of ϵ (Figure 6A). The difference between the FID_train_ and FID_test_ can be seen as a measure of the degree to which the images overfit the training set. Even for the model with our largest ϵ = 10.2, the difference between FID_train_ and FID_test_ was only half compared to the difference of the model without any privacy constraints. This shows that differential privacy substantially contributed to the prevention of the memorization of the training set. Those findings are also in line with the embedding shown in Figure 9 in which the differentially private images are further away from the training images compared to the images generated without any privacy guarantees.

Machine learning models including GANs are susceptible to so-called membership inference attacks (Shokri et al., 2017; Hayes et al., 2019; Chen et al., 2020). Here, an attack model is trained to predict whether a sample was part of the training set. If these attacks are successful, the privacy of the training samples is jeopardized. Differential privacy has been shown to decrease the model's vulnerability to privacy attacks (Shokri et al., 2017; Hayes et al., 2019). While there is no consensus about an exact value of ϵ, studies such as Hayes et al. (2019) and Bagdasaryan and Shmatikov (2019) consider a value of ϵ < 10 acceptable. In this study, we were able to synthesize image-label pairs with single-digit ϵ (i.e., ϵ = 7.4) that still show reasonable performance in the segmentation task. Naturally, further research is necessary to validate that our models would successfully defend against membership inference attacks.

Whereas, the segmentation performance in terms of DSC showed a consistent trend, this was not always true for the bAHD. Figure 3C shows overall comparable results to the DSC performance with some fluctuations. These fluctuations can be explained by selecting the best model based on the best validation DSC and not bAHD. In Figure 5B, however, the segmentation model for ϵ = 1.3 seemed to perform better compared to models with ϵ = 1.9 and ϵ = 2.7. An explanation for this might be the number of false positives and false negatives in the segmentations. For ϵ = 1.3, barely any voxel was identified as belonging to a vessel which resulted in many false negatives. For the other two models, there were many false positives with a large distance to the ground truth. The bAHD considers these models to be worse although none of the three models show a good segmentation performance (see Figure S2 in the supplementary material). The characteristic of penalizing especially false positives should be taken into consideration in future studies when using the bAVD as a metric.

The main limitations of the present study are the computational restrictions. Due to that only 2D patches were used. Additionally, more complex GAN architectures consisting of multiple generators and/or discriminators such as PrivGAN (Mukherjee et al., 2021) or PATE-GAN (Yoon et al., 2019) could not be implemented. Especially PrivGAN appears to be an interesting direction for future research since it does not only implement differential privacy but also aims to reduce vulnerability toward membership inference attacks directly.

## 6. Conclusion

In the present study, we synthesized differentially private TOF-MRA images and segmentation labels using GANs for a neuroimaging application. We proposed different evaluation metrics including the performance of a trained neural network for vessel segmentation. Even with privacy constraints, we could train a segmentation model that works reasonably well on real patient data. This is a crucial step toward synthesizing medical imaging data that both preserves predictive properties and privacy. Nonetheless, further studies should be conducted to evaluate if our findings generalize to other types of medical imaging data and to further improve performance. Our synthetic data is available upon request.

## Data Availability Statement

The data analyzed in this study is subject to the following licenses/restrictions: The datasets used in this article are not readily available because data protection laws prohibit sharing the PEGASUS and 1000Plus datasets at the current time point. Requests to access these datasets should be directed to ethikkommission@charite.de.

## Ethics Statement

The studies involving human participants were reviewed and approved by Ethics Committee of Charité University Medicine Berlin and Berlin State Ethics Board. The patients/participants provided their written informed consent to participate in this study.

## Author Contributions

TK, MH, VM, FB, KS, AHe, KH, SP, AHi, and DF: concept and design. VM, JS, IG, AK, and JF: acquisition of data. TK, VM, FB, AHe, KH, and DF: model design. TK: data analysis. TK, MH, VM, FB, AHe, KH, and DF: data interpretation. TK, MH, VM, FB, KS, AHe, KH, SP, AHi, JS, IG, AK, JF, and DF: manuscript drafting and approval. All authors contributed to the article and approved the submitted version.

## Funding

This study has received funding from the European Commission through a Horizon2020 grant (PRECISE4Q grant no. 777 107, coordinator: DF) and the German Federal Ministry of Education and Research through a Go-Bio grant (PREDICTioN2020 grant no. 031B0154 lead: DF).

## Conflict of Interest

TK, MH, VM, and AHi are employed by ai4medicine. FB and AHe are employed by Fraunhofer. JS reports receipt of speakers' honoraria from Pfizer, Boehringer Ingelheim, and Daiichi Sankyo. JF has received consulting and advisory board fees from BioClinica, Cerevast, Artemida, Brainomix, Biogen, BMS, EISAI, and Guerbet. DF receiving grants from the European Commission, reported receiving personal fees from and holding an equity interest in ai4medicine. The remaining authors declare that the research was conducted in the absence of any commercial or financial relationships that could be construed as a potential conflict of interest.

## Publisher's Note

All claims expressed in this article are solely those of the authors and do not necessarily represent those of their affiliated organizations, or those of the publisher, the editors and the reviewers. Any product that may be evaluated in this article, or claim that may be made by its manufacturer, is not guaranteed or endorsed by the publisher.

## Footnotes

^1^ https://github.com/pytorch/opacus/blob/master/examples/dcgan.py

^2^ https://github.com/prediction2020/Labeled-TOF-MRA-with-DP
