# Supplementary material for: Toward Sharing Brain Images: Differentially Private TOF-MRA Images With Segmentation Labels Using Generative Adversarial Networks
Source: Front Artif Intell. 2022 May 2;5:813842. doi: 10.3389/frai.2022.813842 (PMC9108458; doi:10.3389/frai.2022.813842)
Supplement: Supplementary file 1 [file Data_Sheet_1.PDF]

Supplementary Material

1 SUPPLEMENTARY DATA

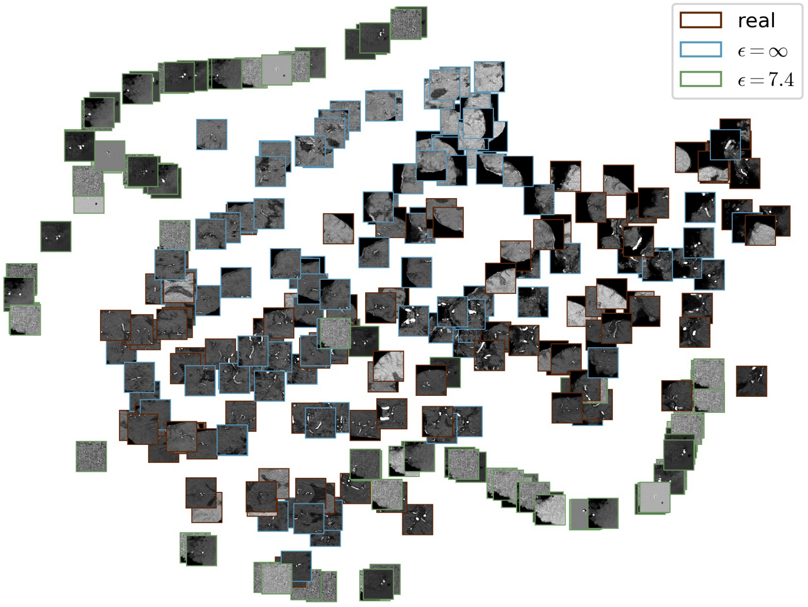

Figure S1. Visualization of real and generated images with and without differential privacy in a t-SNE embedding. The distribution of real images and generated images without privacy almost entirely overlap. In contrast to that, the images with privacy guarantees are only partly overlapping and cluster at the edges, distant from the real images.

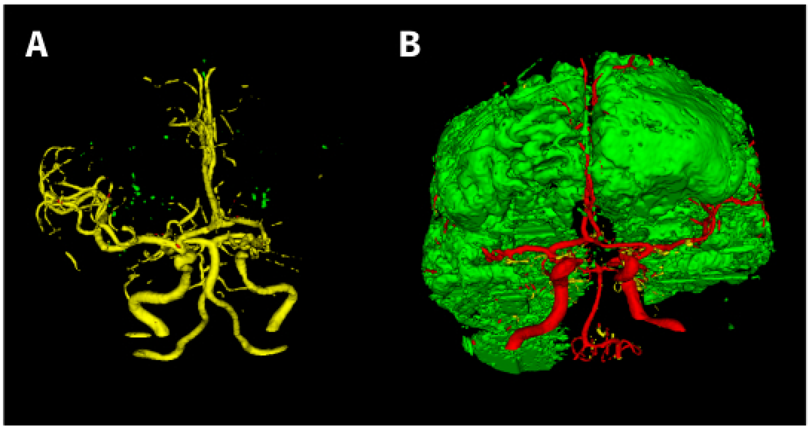

Figure S2. Segmentation error maps for two example patients for a model with $\epsilon = 1.3$ **(A)** and $\epsilon = 2.7$ **(B)**. Voxels in red are true positives, yellow represents false negatives and green false positives. **(A)** shows many false negatives with few false positives. The Dice Similarity Coefficient (DSC) is 0.046 and the balanced average Hausdorff distance (bAHD) 8.3. **(B)** shows many false positives with a DSC of 0.052 and a bAHD of 190.5.
